# Determination of structural constraint within the HIV proteome through analysis of amino acid microenvironments

**DOI:** 10.1186/1742-4690-9-S2-P349

**Published:** 2012-09-13

**Authors:** G Gaiha, M Anahtar, E Rossin, B Walker

**Affiliations:** 1 Ragon Institute, Charlestown, MA, USA; 2 Broad Institute, Boston, MA, USA

## Background

The design of an effective T cell based vaccine relies on determining the most highly constrained regions of the HIV proteome.

## Methods

Using publicly available crystal structures from the Protein Databank (PDB), we performed a systematic analysis of the local microenvironment of every amino acid within the HIV Proteome for which structural data was available (65.8%). Structural constraint parameters included involvement in protein secondary structure, relative solvent accessibility, and involvement of amino acid side chains in intermolecular interactions (Van der Waals bonds, hydrogen bonding, salt bridges, disulfide bridges, pi-pi bonds, and pi-cation bonds). Calculations of these constraints were carried out using validated methods of protein structure analysis and distance geometry, with weighted values cumulatively summed to provide a constraint score for every amino acid.

## Results

Amino acids with a higher constraint score were observed to strongly correlate with low values of entropy within viral sequences from every clade of HIV. Analysis of constraint score variation across the HIV proteome reveals that the p24 capsid protein to be the most highly interconnected and constrained. Evaluation of amino acids within known HLA-restricted epitopes further elucidated a preference of controller alleles for buried and interconnected amino acids, while progressor alleles predominantly targeted exposed and non-connected amino acids. Thermodynamic stability analysis further demonstrated a strong correlation with amino acid constraint and change in predicted Gibbs' Free Energy.

## Conclusion

Our analyses reveal that evaluation of local amino acid microenvironments represents a novel method for the determination of constraint within the HIV proteome, setting the stage for more robust targeting of these constrained regions and enhanced immunogen design.

